# Spiral Antenna-Coupled Microbridge Structures for THz Application

**DOI:** 10.1186/s11671-017-1857-7

**Published:** 2017-02-06

**Authors:** Jun Gou, Tian Zhang, Jun Wang, Yadong Jiang

**Affiliations:** 1 State Key Laboratory of Electronic Thin Films and Integrated Devices, University of Electronic Science and Technology of China, Chengdu, 610054 China; 2 School of Optoelectronic Information, University of Electronic Science and Technology of China, Chengdu, 610054 China

**Keywords:** THz, Spiral antenna, Microbolometer, Design, Absorption

## Abstract

Bolometer sensor is a good candidate for THz imaging due to its compact system, low cost, and wideband operation. Based on infrared microbolometer structures, two kinds of antenna-coupled microbridge structures are proposed with different spiral antennas: spiral antenna on support layer and spiral antenna with extended legs. Aiming at applications in detection and imaging, simulations are carried out mainly for optimized absorption at 2.52 THz, which is the radiation frequency of far-infrared CO_2_ lasers. The effects of rotation angle, line width, and spacing of the spiral antenna on THz wave absorption of microbridge structures are discussed. Spiral antenna, with extended legs, is a good solution for high absorption rate at low absorption frequency and can be used as electrode lead simultaneously for simplified manufacturing process. A spiral antenna-coupled microbridge structure with an absorption rate of more than 75% at 2.52 THz is achieved by optimizing the structure parameters. This research demonstrates the use of different spiral antennas for enhanced and tunable THz absorption of microbridge structures and provides an effective way to fabricate THz microbolometer detectors with great potential in the application of real-time THz imaging.

## Background

The unique spectral characteristics of terahertz (THz) radiation (0.3~10 THz), such as a good transparency of non-polar materials including cloths, envelopes and plastic packages, high reflection on metals, and very low risk for health concern, make it attractive for sensing and imaging applications including safety inspection for concealed objects [1, 2], medical diagnosis, and non-destructive testing of materials [3]. THz imaging is currently conducted primarily through the use of semiconductor detectors, such as Schottky diodes [4] and field effect transistors [5], and thermal detectors such as superconducting hot spot bolometers [6] and room temperature microbolometers. Microbolometers produce images by detecting bending of microcantilever based on surface plasmon resonance [7–9], or resistance change of thermal sensitive film [10–12] caused by temperature change of the sensing element due to incident radiation absorption. Compared to other detection schemes, microbolometer detectors have a broad wavelength response from infrared to millimeter wave band and, unlike photon-based detector, can be operated at room temperature. Since infrared (IR) uncooled microbolometer, focal-plane array has been developed for years and a series of products are supplied by institutes including Raytheon [13, 14], BAE [15], and DRS [16]. The thermal conversion mechanism allows one to shift the microbolometer response to THz range and the mature infrared technology including manufacturing process, and readout procedures provide a good technical foundation. However, such IR detectors with traditional microbridge structures exhibit limited sensitivity for THz detection due to poor absorption of THz radiation [17, 18]. Aimed at this problem, some improvements have been made on microbridge structures and membrane materials. A double-layer structure is introduced in [19] to obtain high fill factor. A thin metamaterial film tuned to the illuminator frequency is integrated in [20]. Nanostructured metallic thin film absorber is integrated in [21] by a combined process of magnetron sputtering and reactive-ion etching (RIE) for enhanced THz absorption due to increased specific surface area.

In order to increase the absorbed power, another effective method is to introduce an antenna-coupled structure. The antenna receives the electromagnetic wave and the received energy is directly coupled to the microbolometer for maximum energy collection [22–25]. Starting with infrared microbolometer technology, highly sensitive uncooled antenna-coupled microbolometer focal-plane arrays have been developed at CEA-Leti based on an innovative use of antennas and an optimization of resonant cavity [26, 27]. The design of antenna-coupled microbolometer mainly aims at increasing its gain–bandwidth product and minimizing its thermal mass for fast frame rates [28]. However, the heating rate of an antenna decreases with the increase of its gain–bandwidth product due to increased bulk volume. O. Markish and Y. Leviatan take into account both the thermal and electromagnetic aspects when designing the antenna and suggest that wire antennas such as dipole and bow tie antennas, albeit not necessarily best in terms of gain–bandwidth product, are preferable over planar antennas for a typical heating rate requirement [29]. Considering the structure characteristics of microbolometers, this paper proposed two kinds of novel antenna-coupled microbridge structures with different spiral antennas: spiral antenna on support layer and spiral antenna with extended legs. Aiming at applications in detection and imaging, simulations were carried out mainly for optimized absorption of 2.52 THz wave radiated by high-power far-infrared CO_2_ gas laser. The influences of different structure parameters of the spiral antenna on THz wave absorption were studied. A spiral antenna-coupled microbridge structure suitable for terahertz wave detection was obtained by optimizing the structure parameters.

## Results and Discussion

### Microbridge Structure with Spiral Antenna on Support Layer

**Fig. 1:**
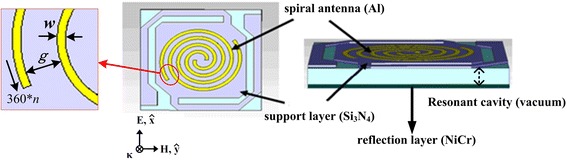
Spiral antenna-coupled microbridge structure

The 35-μm pitch pixel of THz microbolometer detector with a microbridge structure, shown in Fig. 1, was composed of diaphragm (sensitive area), cell contact, and two L-type legs which supported the diaphragm. This structure had a long leg length and a large sensitive area. The diaphragm consisted of support layer, thermal sensitive layer (vanadium oxide), and THz absorption layer, with a reflection layer placed 2 μm away. The signal of the diaphragm was transferred via the cell contact to readout integrated circuit (ROIC) located under the reflection layer. Spiral antenna structure acting as the THz absorption layer was integrated on the top of the support layer with a size of about 24 × 24 μm. In our antenna-coupled microbridge structure, 0.1-μm nickel–chromium (NiCr) thin film acted as the reflection layer and 0.4-μm silicon nitride (Si_3_N_4_) film acted as the support layer. Spiral antenna structure was made of aluminum (Al) with a thickness of 0.1 μm and an outside diameter of 20.8 μm. The effects of structure parameters of spiral antenna on THz wave absorption of microbridge structure, including rotation angle (360*n), line width (*w*), and spacing (*g*) which are indicated in Fig. 1 with a partial enlarged drawing of the spiral antenna, were studied by changing different structure parameters.

#### Effect of Rotation Angle

**Fig. 2:**
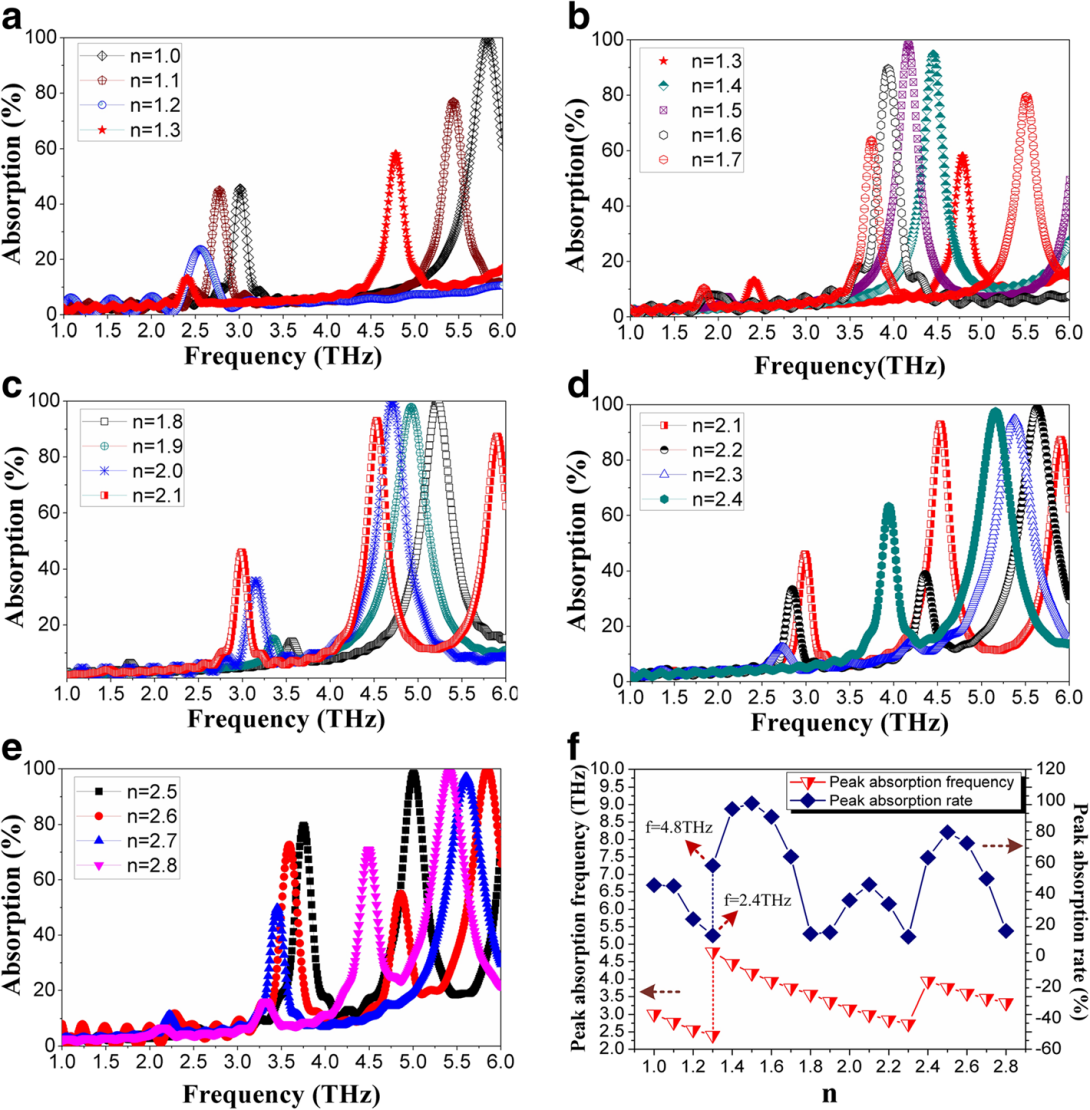
THz wave absorption curves of microbridge structures with different rotation angles (360*n) of spiral antenna: **a** *n* = 1.0~1.3; **b** *n* = 1.3~1.5; **c** *n* = 1.5~1.7; **d** *n* = 1.8~2.1; **e** *n* = 2.1~2.4; and **f** *n* = 2.5~2.8

The effect of rotation angle of the spiral antenna was first studied by fixing the line width to 1 μm and setting the rotation angle to 360*n (the rotation angle starting from the center of the spiral antenna, *n* changed in 1~2.8) in the simulation. The THz wave absorption curves of microbridge structures with different rotation angles of spiral antennas are shown in Fig. 2.

It can be seen from Fig. 2a that the peak absorption rate and the absorption frequency near 3 THz decrease with the increase of rotation angle when *n* = 1.0~1.3. Figure 2b suggests that when *n* = 1.3~1.7, there is a very weak absorption near 3 THz. The peak absorption frequency at higher frequency decreases with the increase of rotation angle. The peak absorption rate increases when *n* = 1.3~1.5 and then decreases when *n* = 1.5~1.7 due to increasing rotation angle. Figure 2c indicates that the peak absorption rate increases and the absorption frequency decreases near 3 THz with the increase of rotation angle when *n* = 1.8~2.1. Figure 2d, e shows the THz wave absorption curves of microbridge structures with different rotation angles (360*n) when *n* = 2.1~2.4 and *n* = 2.5~2.8, respectively. It is clear that the effects of rotation angle on THz wave absorption when *n* = 2.1~2.3, *n* = 2.3~2.5, and *n* = 2.5~2.8 are similar to the effects when *n* = 1~1.3, *n* = 1.3~1.5, and *n* = 1.5~1.8, respectively. This can also be concluded from Fig. 2f, which shows the variation of peak absorption rate and absorption frequency when *n* changes in 1~2.8. It seems that the effect of rotation angle on THz wave absorption has a certain repeatability between *n* = 1~2 and *n* = 2~3.

In addition to the repeatability, Fig. 2 also suggests that the absorption frequency keeps always decreasing with the increase of rotation angle. When *n* = 1.1 and *n* = 2.1, relatively high-peak absorption rate and low absorption frequency are obtained.

#### Effect of Line Width

**Fig. 3:**
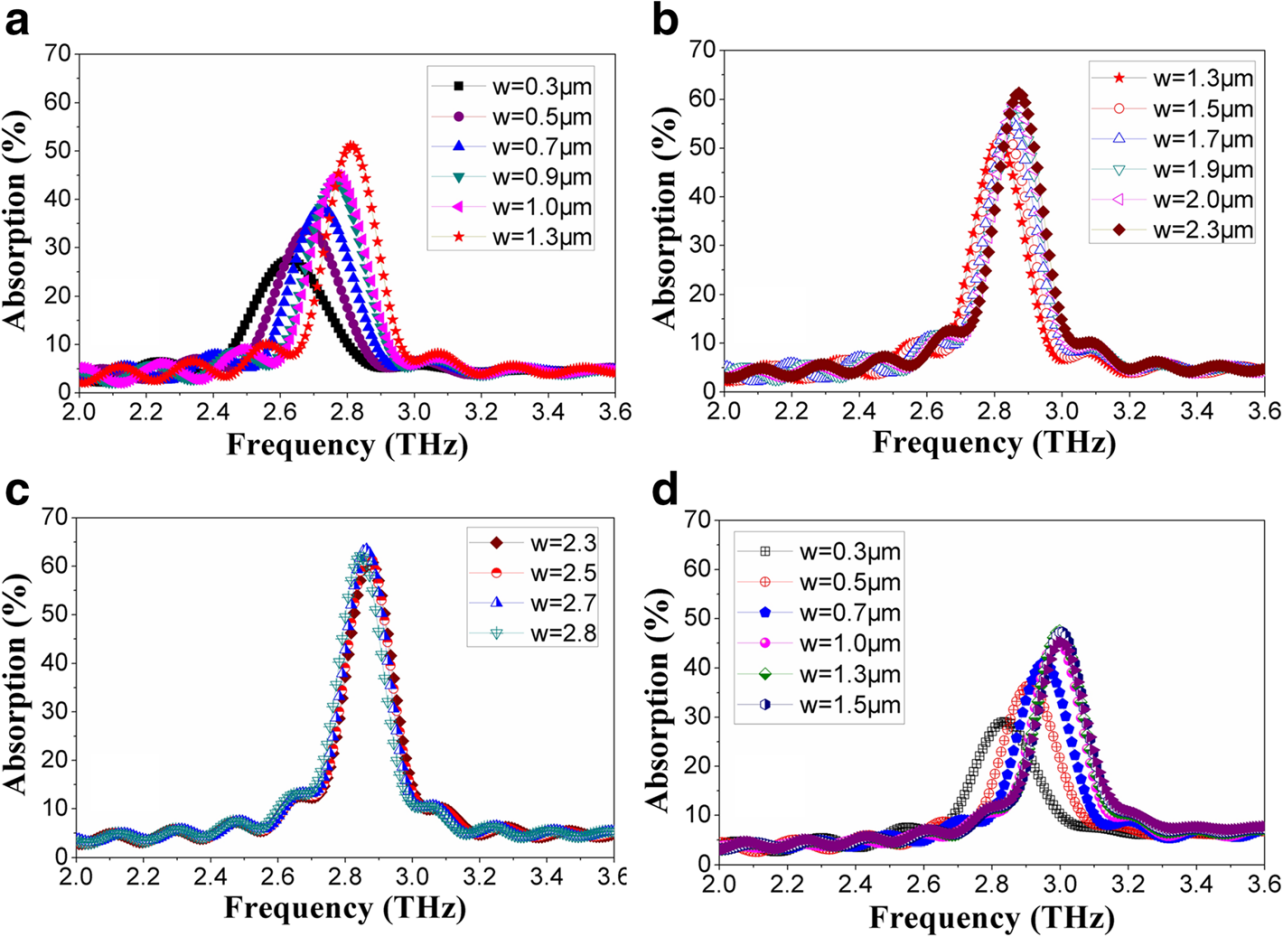
THz wave absorption curves of microbridge structures with different rotation angles (360*n) and line widths (*w*) : **a** *n* = 1.1, *w* = 0.3~1.3; **b** *n* = 1.1, *w* = 1.3~2.3; **c** *n* = 1.1, *w* = 2.3~2.8 ; and **d** *n* = 2.1, *w* = 0.3~1.5

THz wave absorptions of microbridge structures with different line widths (*w*) of the spiral antennas were simulated when *n* = 1.1 (Fig. 3a–c) and *n* = 2.1(Fig. 3d), respectively. Figure 3a shows that the peak absorption rate and absorption frequency increase with the increase of line width in 0.3~1.3 μm. When the line width increases to 1.4~2.3 μm, the increase of peak absorption rate and absorption frequency slow down, as shown in Fig. 3b. When the line width changes in 2.4~2.8 μm (Fig. 3c), the peak absorption rate and absorption frequency remain essentially the same. Similar phenomenon can be observed in Fig. 3d when *n* = 2.1 and *w* = 0.3~1.7 μm, which indicates that the peak absorption rate and absorption frequency remain unchanged when *w* is more than 1.3 μm. This is conducive to the fabrication of spiral antenna-coupled microbridge structure for the slight deviation of line width in patterning will not lead to obvious change in THz wave absorption characteristics of the microbridge structure, which reduces the manufacturing difficulty and improves the process compatibility.

Compared to the spiral antenna with a rotation angle of 360*n (*n* = 2.1), the spiral antenna with a rotation angle of 360*n (*n* = 1.1) has an obvious advantage in that it provides higher peak absorption rate and lower absorption frequency, besides, its simpler structure makes it easier to adjust the parameters of spiral antenna such as line width.

**Fig. 4:**
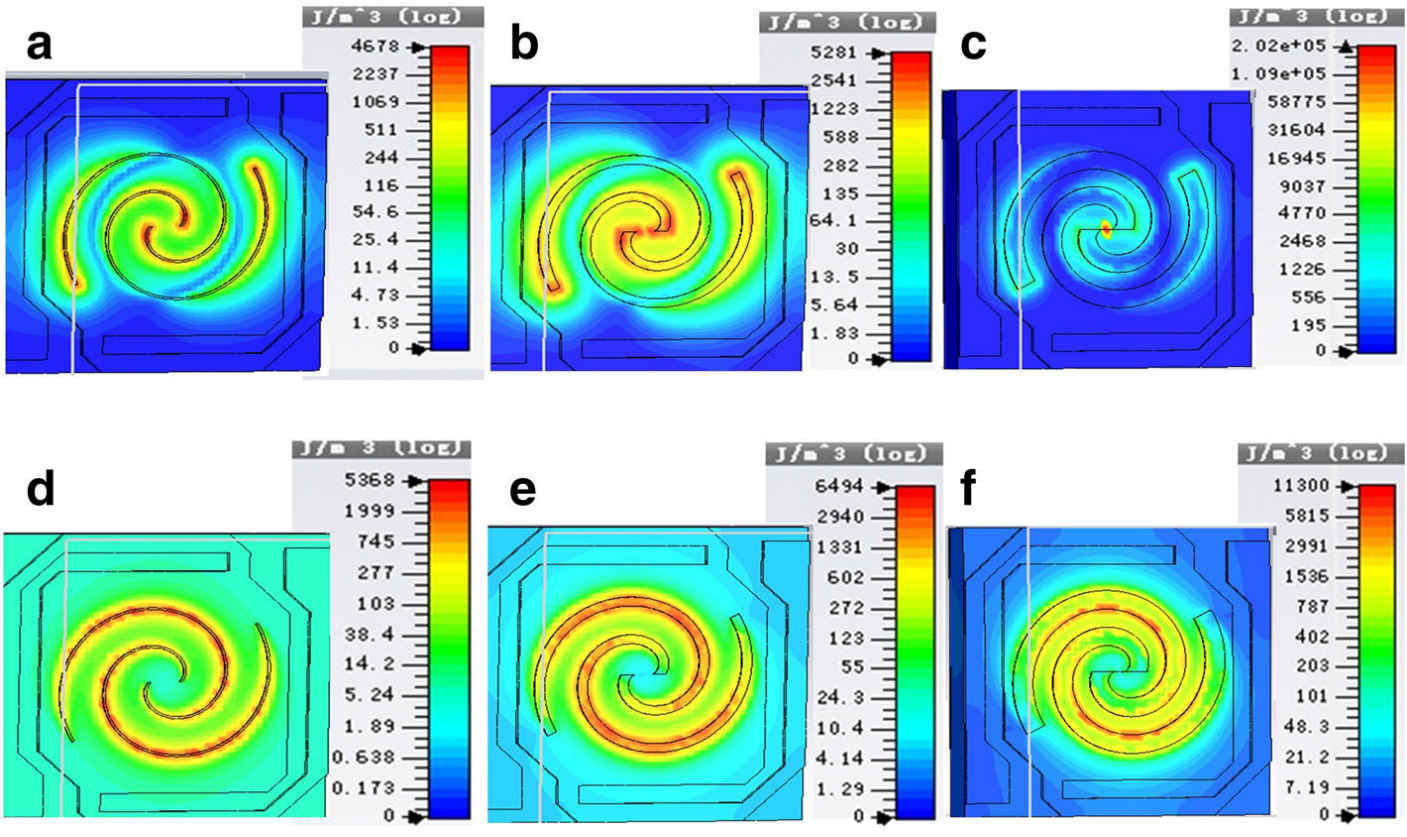
Energy density diagrams of electric field and magnetic field of spiral antenna-coupled microbridge structures when *n* = 1.1 with different line widths (*w*): **a** *w* = 0.3, electric field; **b** *w* = 1.3, electric field; **c** *w* = 2.8, electric field; **d** *w* = 0.3, magnetic field; **e** *w* = 1.3, magnetic field; and **f** *w* = 2.8, magnetic field

Figure 4 shows the energy density diagrams of electric field and magnetic field of spiral antenna-coupled microbridge structures when *n* = 1.1 with line widths of *w* = 0.3 μm, *w* = 1.3 μm, and *w* = 2.8 μm, respectively.

In the energy density diagrams of electric field and magnetic field in Fig. 4, the change of color from blue to red indicates that the absorption rate of terahertz wave becomes larger. Energy of electric field and magnetic field absorbed by the antenna can be converted into heat energy. It can be seen from Fig. 4 that the absorption area of electric field energy is mainly distributed in the center and both ends of the spiral antenna, while the absorption area of magnetic field energy is mainly located in the position of antenna line. With the increase of line width, the absorption area of electric field energy moves toward the center and both ends and becomes more concentrated. The distribution of absorption area of magnetic field energy becomes more uniform due to the increase of line width.

#### Effect of Spacing

**Fig. 5:**
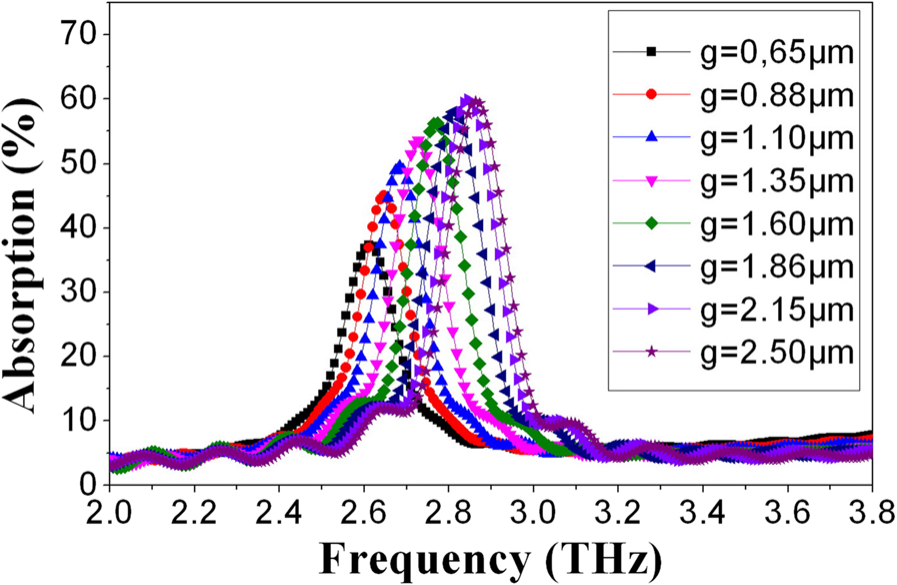
THz wave absorption curves of microbridge structures with different spacing (*g*)

THz wave absorption curves of microbridge structures (*n* = 1.1, *w* = 2 μm) with different spacing (spacing between adjacent lines, *g* = 0.65~2.5) are shown in Fig. 5. Figure 5 suggests that the peak absorption rate and absorption frequency increase with the increase of spacing from 0.65 to 2.5 μm. The results in Fig. 5 show no linear variation of the peak absorption rate and absorption frequency with the increasing of spacing. As the spacing increases, the increase of peak absorption rate and absorption frequency becomes slower.

As we have discussed before, the increase of peak absorption rate and absorption frequency slows down with the increase of line width and remains unchanged when the line width is bigger than a certain value. Now, it becomes clear that, this effect is not only attributed to the change of line width, the increase of line width will lead to the decrease of spacing in the case of constant outside diameter of spiral antenna. The peak absorption rate and absorption frequency increase with the increase of line width, while it has opposite change trend with the increase of spacing.

The effects of different structure parameters of spiral antenna on THz wave absorption of microbridge structure have been discussed by the simulation before. Some interested results are obtained. However, the absorption rate and absorption frequency have not reached the ideal value for the target frequency of 2.52 THz, and it seems difficult to achieve due to the size of spiral antenna limited by the size of the support layer.

### Spiral Antenna with Extended Legs

**Fig. 6:**
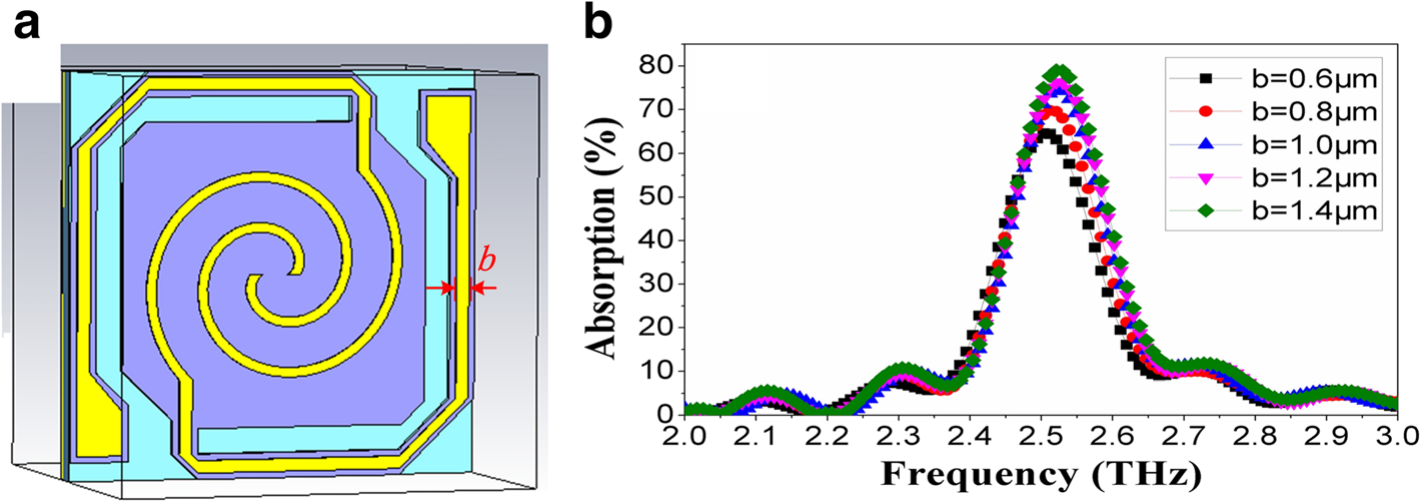
**a** Novel spiral antenna structure with legs and **b** THz wave absorption curves with different leg widths (*b*)

Aimed at the limitation of the size of the support layer, a novel spiral antenna-coupled microbridge structure was proposed by making full use of the structure characteristics of the microbolometer. As shown in Fig 6a, the spiral antenna is extended to the bridge legs to increase the effective area of the antenna.

THz wave absorption curves of spiral antenna-coupled microbridge structures (*n* = 1.1, *w* = 1 μm) with different leg widths (*b* = 0.6~1.4) are shown in Fig. 6b. Figure 6b indicates that the absorption frequency is decreased to near 2.52 THz and the peak absorption rate is improved obviously due to the contribution of the extended legs. The peak absorption rate and absorption frequency increase with the increase of leg width.

**Fig. 7:**
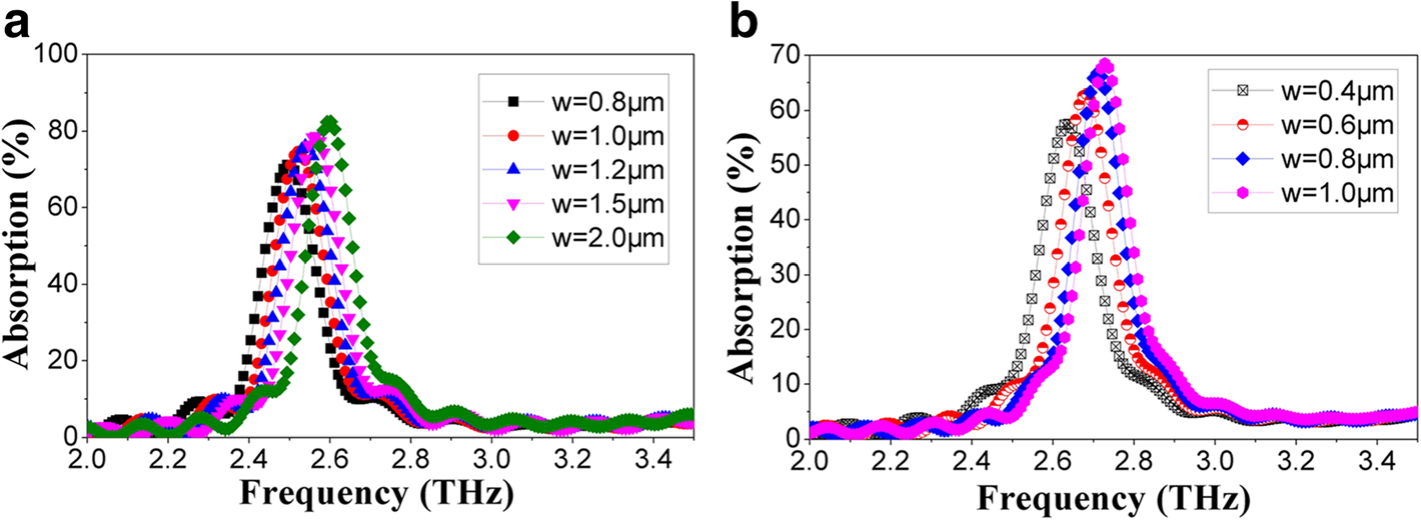
THz wave absorption curves of microbridge structures with a leg width of 1 μm when *n* = 1.1(**a**) and *n* = 2.1 (**b**) with different line widths (*w*)

THz wave absorptions of microbridge structures with different line width (*w*) of the spiral antenna with a leg width of 1 μm were simulated when *n* = 1.1 (Fig. 7a) and *n* = 2.1(Fig. 7b), respectively. Since the outside diameters of spiral antenna structures were limited by the size of the support layer (about 24 × 24 μm) and spiral antenna with a greater rotation angle permits a smaller line width in the case of the same outside diameter (20.8 μm), the line widths (*w*) of the spiral antennas with different rotation angle (360*n) were set to 0.8~2.0 and 0.4~1.0 μm when *n* = 1.1 and *n* = 2.1, respectively. Figure 7 shows that the peak absorption rate and absorption frequency increase with the increase of line width both when *n* = 1.1 and *n* = 2.1. It becomes clear that the extending of spiral antenna contributes to the decrease of absorption frequency. It can be seen that peak absorption of spiral antenna-coupled microbridge structure occurs at 2.52 THz when *n* = 1.1 with a line width of 1 μm and a leg width of 1 μm, and an absorption rate of more than 75% is achieved. Figure 7b suggests that the absorption frequency can hardly be optimized to near 2.52 THz when *n* = 2.1 for a line width smaller than 0.4 μm will be required, which results in difficulty in manufacturing.

**Fig. 8:**
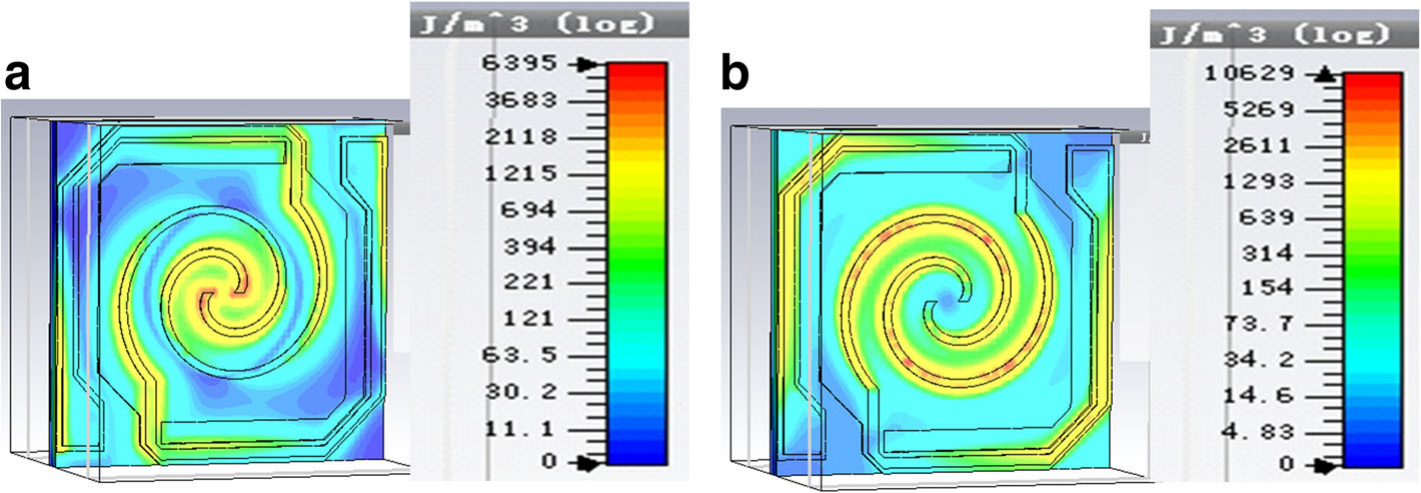
Energy density diagrams of electric field (**a**) and magnetic field (**b**) of spiral antenna-coupled microbridge structure when *n* = 1.1 with a line width of 1 μm and a leg width of 1 μm

Figure 8 shows the energy density diagrams of electric field and magnetic field of spiral antenna-coupled microbridge structures when *n* = 1.1 with a line width of 1 μm and a leg width of 1 μm. It can be seen that THz wave is absorbed by both spiral antenna and the legs, while spiral antenna on the support layer makes greater contribution, which is advantageous to temperature rise of the diaphragm with thermal sensitive film.

The design of novel spiral antenna with extended legs is a good solution for high absorption rate at low absorption frequency of 2.52 THz. At the same time, the spiral antenna is extended to the positions of cell contacts through the bridge legs. This design makes it possible to use the spiral antenna as electrode lead simultaneously to connect the thermal sensitive film to readout circuit, which is very beneficial to integration and process simplification. This provides an effective way which is easy to accomplish and compatible with the manufacturing process of THz microbolometer focal-plane array to enhance THz absorption and improve detection performance.

## Conclusions

In this paper, we have presented the design and electromagnetic simulation of antenna-coupled bolometer that works in THz band. Starting with infrared microbolometer technology, spiral antenna-coupled microbridge structures were developed in this paper. Novel spiral antenna with extended legs was first designed based on the structure characteristics of microbolometer. Results of the structure design and electromagnetic simulation were presented, concentrating on the spectral absorption. The influences of different structure parameters of the spiral antenna on THz wave absorption were discussed. The extended legs of spiral antenna, which contributed to the increase of THz wave absorption rate and the decrease of absorption frequency, made it possible to use the spiral antenna as electrode lead simultaneously for process simplification. A spiral antenna-coupled microbridge structure with optimized absorption at 2.52 THz was obtained, aiming at applications in detection and imaging. This research was only the first step in demonstrating the feasibility of antenna-coupled THz bolometer detectors by proposing novel spiral antenna-coupled microbridge structures and demonstrating its improvements on THz absorption and manufacturing process. However, it established a foundation for fabrication of THz microbolometer and its applications in sensing and imaging which will be done in further work.

## Methods

To explain the nature of the frequency tuning by spiral antennas, finite element simulations were carried out using commercial software Microwave Studios by CST. We simulated a single unit cell of antenna-coupled microbridge structure as shown in Fig. 1. The Al regions were simulated as lossy metal with a conductivity of *σ*_Al_ = 3.56 × 10^7^S/m, and the reflection layer was modeled as lossy NiCr with *σ*_NiCr_ = 1 × 10^7^S/m. The Si_3_N_4_ layer supporting the antenna was modeled as a dielectric with ${\varepsilon}_{{\mathrm{Si}}_3{\mathrm{N}}_4}=1$ and ${\sigma}_{\mathrm{S}{\mathrm{i}}_3{\mathrm{N}}_4}=0\mathrm{S}/\mathrm{m}$, and the resonant cavity underlying the diaphragm was modeled with *ε*_vacuum_ = 1 and *σ*_*vacuum*_ = 0S/m. In simulations, we used adaptive mesh refinement to ensure an accurate numerical solution with a short simulation time. The final mesh typically contained about 93,000 hexahedrons, and the minimum edge length was about 0.15 μm. We investigated the S-parameters of transmission $\left({\tilde{S}}_{21}\right)$ and reflection $\left({\tilde{S}}_{11}\right)$ of a single unit cell with perfect electric (PE) and perfect magnetic (PM) boundary conditions along the $\widehat{x}$ and *ŷ* directions, respectively. The absorptivity was calculated using the equation *A* = 1 − |*S*_21_|^2^ − |*S*_11_|^2^.

## Abbreviations

IR: Infrared
NiCr: Nickel–chromium
PE: Perfect electric
PM: Perfect magnetic
RIE: Reactive-ion etching
ROIC: Readout integrated circuit
Si_3_N_4_: Silicon nitride
THz: Terahertz
